# A novel mutation in gelatinous drop-like corneal dystrophy and functional analysis

**DOI:** 10.1038/s41439-019-0060-z

**Published:** 2019-07-11

**Authors:** Yukiko Nagahara, Motokazu Tsujikawa, Toru Takigawa, Peng Xu, Chifune Kai, Satoshi Kawasaki, Mina Nakatsukasa, Tsutomu Inatomi, Shigeru Kinoshita, Kohji Nishida

**Affiliations:** 1 Department of Ophthalmology, Osaka University Graduate School of Medicine, Suita, Osaka Japan; 2 Department of Biomedical Informatics, Osaka University Graduate School of Medicine, Suita, Osaka Japan; 3 Department of Ophthalmology, Kyoto Prefectural University of Medicine, Kyoto, Japan

**Keywords:** Gene expression, Genetics

## Abstract

We identified a novel mutation of the *tumor-associated calcium signal transducer 2* (*TACSTD2*) gene in a Japanese patient with gelatinous drop-like corneal dystrophy (GDLD). Genetic analysis revealed a novel homozygous mutation (c.798delG, which may result in frameshift mutation p.Lys267SerfsTer4) in the *TACSTD2* gene. This mutated gene was devoid of its original function in helping the claudin (CLDN) 1 and 7 proteins transfer from the cytoplasm to the plasma membrane.

## Eye disorder: Novel mutation linked to rare corneal disease

DNA sequencing of a gene linked to gelatinous drop-like corneal dystrophy (GDLD) has revealed a novel disease-causing mutation responsible for the rare hereditary eye disorder. A team from Japan led by Motokazu Tsujikawa from Osaka University treated a 44-year-old man with vision problems. The characteristic grayish deposits of amyloid protein in the otherwise clear front surface of the man’s eyes led the researchers to suspect the patient had GDLD. Analysis of the *TACSTD2* gene, which is often mutated in affected individuals, revealed two copies of the same tiny deletion, a mutation that caused both gene copies to encode a shortened protein. Cell experiments showed that the defective protein could no longer bind and properly position other proteins within the cornea to form the outer epithelial barrier. The findings could aid in future diagnoses of GDLD.

## Introduction

Gelatinous drop-like corneal dystrophy (GDLD; OMIM:204870) is a rare corneal dystrophy. Most affected patients are Japanese, and the estimated incidence is 1/33,000 in Japan^1^. GDLD is an autosomal recessive disease characterized by the deposition of amyloid in the subepithelial region of the bilateral corneas. As amyloid deposition increases and corneal neovascularization covers the corneal surface, visual acuity becomes severely impaired. Repeated lamellar or penetrating keratoplasty is frequently required for most patients. Using positional cloning, we successfully identified the disease-causing gene, *tumor-associated calcium signal transducer 2* (*TACSTD2*;NM_002353), thereby enabling us to investigate the molecular bases of GDLD^2–4^. To date, 31 different GDLD-causing alterations of the *TACSTD2* gene (11 missense, 7 nonsense, and 13 frameshift mutations) have been reported to our knowledge^5–16^. In this study, we identified a novel homozygous frameshift mutation in the *TACSTD2* gene in a Japanese family with GDLD and evaluated the pathogenic effect of the mutation.

## Materials and methods

All experimental procedures for the sequencing analysis were approved by the Institutional Review Board for Human Studies at Kyoto Prefectural University of Medicine (approval number RBMR-G-148-1). All experimental procedures for the functional analysis were approved by the Institutional Review Board for gene recombination at Osaka University (approval number 2973). Prior informed consent was obtained from the investigated pedigree member after a detailed explanation of the study protocols, and this study was performed in accordance with the tenets of the Declaration of Helsinki for research involving human subjects.

Genomic DNA was extracted from peripheral blood. Polymerase chain reaction (PCR) was performed with a primer pair against *TACSTD2* (M1S1-F-2; 5′-CCT GCA GAC CAT CCC AGA C-3′, M1S1-R-2; 5′-CAG GAA GCG TGA CTC ACT TG-3′), which fully covered the coding region of this gene. The PCR product was purified and bidirectionally sequenced using a Big-Dye Terminator v3.1 cycle sequencing kit (Applied Biosystems, Inc., Foster City, CA) in a 20 μl reaction buffer containing a 2× sequencing mixture and either of the above primers. After ethanol precipitation, the sequence products were electrophoresed on an automated capillary sequencer (Genetic Analyzer; Applied Biosystems). To further confirm the mutation detected by the above sequencing analysis, primer extension analysis was performed using a commercial kit (Applied Biosystems, Inc.) for the 798th nucleotide of the *TACSTD2* gene coding region in the normal volunteer and patient with the forward primer.

We constructed lentivirus plasmid vectors that harbor the coding region of the *CLDN1* or *CLDN7* gene. Then, we coinfected HeLa cells not expressing the CLDN1, CLDN7 and TACSTD2 proteins with those lentivirus vectors and used the Tet-on system to overexpress the wild-type or mutant *TACSTD2* gene in an inducible manner. First, HeLa cells were seeded at a density of 2.5 × 10^5^　cells per well in a six-well plate and infected with the lentivirus vector expressing the *CLDN1* or *CLDN7* gene. Four days after the infection, drug selection was performed with 0.5 µg/ml puromycin for 2 weeks. Second, we coinfected those HeLa cells with the lentivirus vector expressing the wild-type or mutated *TACSTD2* gene and plenti3.3/TR. Drug selection was performed with 2 µg/ml blasticidin and 500 µg/ml G418 for 2 weeks. The drug-selected HeLa cells, which should express the *CLDN1* or *CLDN7* genes under the control of the CMV promoter as well as the *TACSTD2* gene under the control of a tetracycline-inducible promoter, were seeded on a collagen-coated culture slide (Nunc 177402 Lab-Tek Chamber Slide System with Cover Glass Slide Sterile, Thermo Fisher Scientific Inc.) at a density of 1 × 10^4^ cells per well. Twenty-four hours after seeding, the cells were induced with 1 μg/ml tetracycline for 24 h. After the induction, the cells were fixed with 4% paraformaldehyde, counterstained with Hoechst 33342 dye and mounted with a commercial mounting medium (ProLong® Gold Antifade Mountant, Thermo Fisher Scientific Inc.). The cells were examined under a fluorescent confocal microscope (ELYRA S.1 / LSM710, Carl Zeiss, Oberkochen, Germany) and photographed under the Tet-on system to overexpress the wild-type or mutant *TACSTD2* gene.

**Fig. 1:**
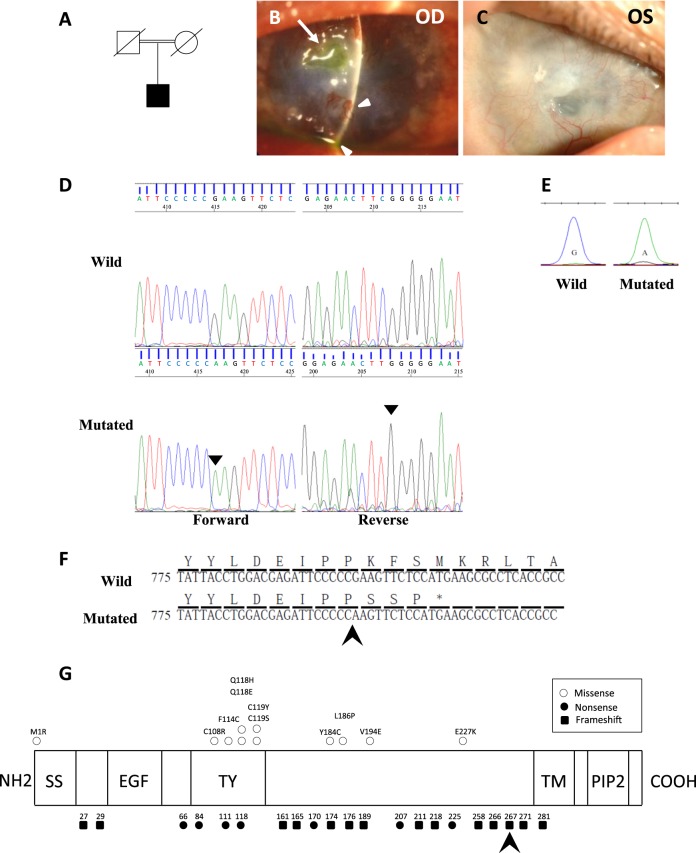
Phenotype and mutation analysis. **a** Family tree of the proband. His parents are a first cousin marriage. He has no brothers. **b** In the left eye of the patient (at age 44), we found a typical mulberry-type GDLD cornea with mulberry depositions and neovascularization in the host cornea (white arrowhead) and an ulcer in the graft cornea (white arrow) before our first operation. **c** In the right eye of the patient (at age 44), remarkable neovascularization was observed and the graft cornea was almost covered by invading conjunctival epithelium with less than 1/4 of the corneal epithelium remaining. Presumably loss of corneal limbal stem cells caused the conjunctival invasion. **d** Results of direct sequencing analysis for *TACSTD2* in a normal volunteer (upper) and the patient with the mutated protein (lower) sequenced in the forward (left) and reverse (right) directions are presented. Arrowheads indicate the homozygous c.798delG mutation. **e** One-base primer extension analysis was used to confirm the identity of the 798th nucleotide of *TACSTD2* in the normal volunteer and patient. **f** Nucleotide and amino acid sequences of the wild-type (upper) and mutated (lower) *TACSTD2* gene on both sides of the c.798delG mutation are shown. **g** Schematic representation of the distribution of reported *TACSTD2* mutations and the domain structure of the TACSTD2 protein. An arrow indicates the 798delG mutation reported here. Missense (open circles) mutations are shown above, and nonsense (filled circles) and frameshift (filled squares) mutations are shown below. SS signal sequence, EGF epidermal growth factor-like domain, TY thyroglobulin-like domain, TM transmembrane domain, PIP2 phosphatidylinositol 4, 5-bis phosphate-binding consensus sequence

The patient was a 44-year-old Japanese male at the time of his first admission to our hospital. His parents were first cousins (Fig. 1a). He had already undergone several penetrating keratoplasty (PKP) surgeries prior to admission. Characteristic findings of GDLD, including graft failure, mulberry deposition, and neovascularization, were observed in his left host cornea (Fig. 1b). In his right eye, neovascularization was marked, and the corneal graft was almost covered by the invading conjunctiva (Fig. 1c). He underwent six PKP surgeries, two in his right eye at the ages of 24 and 27 and four in his left eye at the ages of 25, 36, 44, and 50. Currently, his left eye has a best-corrected visual acuity of hand motion, and his right eye is blinded by glaucoma.

## Results

Sequencing analysis of the *TACSTD2* gene revealed that the patient had a novel homozygous deletion of G at the 798th nucleotide position (c.798delG, Fig. 1d). A single-base primer extension analysis of the 798th nucleotide of the *TACSTD2* gene was performed to confirm the above sequence results (Fig. 1e). This mutation caused a premature truncation (p.Lys267SerfsTer4, Fig. 1f) and loss of the transmembrane and PIP2 domains (Fig. 1g). We confirmed that this mutation was not registered in the public SNP databases (ExAC, 1000Genome, HGVD, ToMmo).

In normal corneas, the TACSTD2 protein binds to the CLDN1 and CLDN7 proteins to prevent the degradation of these two molecules. In the absence of functional TACSTD2, the subcellular localization of the CLDN1 and CLDN7 proteins changes from the cell membrane to the intracellular region^17^. Thus, we examined the subcellular localization of CLDN1 or CLDN7 in the presence of wild-type or p.Lys267SerfsTer4 mutated *TACSTD2* using the Tet-on system in HeLa cells that express neither TACSTD2 nor CLDNs. We confirmed the expression of the TACSTD2 protein using the Tet-on system and confirmed cell transfection (data not shown).

**Fig. 2:**
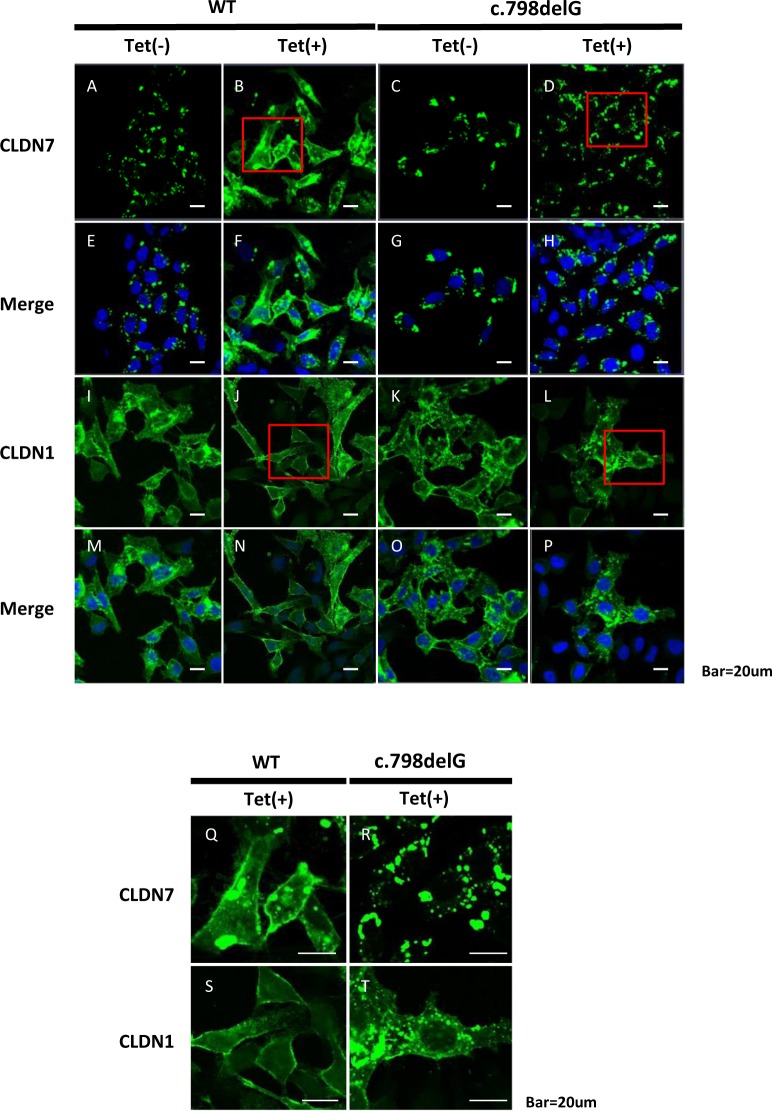
Subcellular localization of CLDNs in cells with the wild-type or p.Lys267SerfsTer4 mutated *TACSTD2* gene. Without *TACSTD2* gene induction, aggregated CLDN7 signals were evident, some of them seems to be in the intracellular organelles (**a**, **c**, **e**, **g**). After induction of wild-type *TACSTD2* gene by tetracyclin, distribution of CLDN7 was spread with more uniformity in cytoplasm and cell membrane (**b**, **f**, **q**). In contrast after induction of p.Lys267SerfsTer4-mutated *TACSTD2* gene, aggregated signal is apparent and the change of subcellular localization of CLDN7 was not significant (**d**, **h**, **r**). CLDN1 protein exhibited almost the same subcellular localization as CLDN7. Without *TACSTD2 gene* induction, CLDN1 signals showed aggregated pattern (**i**, **k**, **m**, **o**). After induction of wild-type *TACSTD2 gene*, distribution of CLDN1 showed more uniformity in cytoplasm (**j**, **n**, **s**). CLDN1 signals were not altered by induction of the mutated *TACSTD2 gene*. The aggregated bodies were apparent (**l**, **p**, **t**)

Without *TACSTD2* expression, we found overexpression of CLDN7 proteins localized at the intracellular region (Fig. 2a, c, e, g). After wild-type *TACSTD2* induction, the distribution of CLDN7 was uniformly spread into the plasma membrane (Fig. 2b, f, q). In contrast, p.Lys267SerfsTer4 *TACSTD2* induction did not alter the subcellular localization of CLDN7 (Fig. 2d, h, r).

The CLDN1 protein exhibited almost the same pattern as the CLDN7 protein. Without *TACSTD2* expression, CLDN1 proteins localized in the intracellular region (Fig. 2i, k, m, o). After wild-type *TACSTD2* induction, CLDN1 spread to the plasma membrane (Fig. 2j, n, s). In contrast, the signals were not altered with the mutated *TACSTD2* (Fig. 2l, p, t).

These results strongly indicate that the p.Lys267SerfsTer4 mutation is deleterious, causing a change in CLDN localization and tight-junction disruption.

## Discussion

The patient demonstrated severe amyloid deposition, corneal neovascularization, and decreased epithelial barrier function, which are the characteristic clinical manifestations of GDLD. Remarkable neovascularization and conjunctival invasion in his right eye occurred presumably as a result of cell cycle acceleration in the limbal cornea. He may present with the typical mulberry-type GDLD^18^, which is not specific to this mutation^19^.

The TACSTD2 protein is a type I single transmembrane protein. The mutation examined was a frameshift mutation that led to premature truncation and loss of the transmembrane domain. Therefore, this frameshift mutation was thought to inactivate the TACSTD2 protein. However, the possibility of errors in translation, for example, due to shifts in the reading frame, shunting of ribosomes, or skipping of stop codons, cannot be denied; therefore, we investigated the subcellular localization of CLDN1 and CLDN7 in HeLa cells. From the functional analysis, we concluded that the mutation in TACSTD2 is indeed pathological.

In conclusion, we report a novel homozygous *TACSTD2* gene mutation (c.798delG that can result in a frameshift, p.Lys267SerfsTer4) in a Japanese patient with GDLD who was born to a consanguineous couple. Our functional study revealed that the mutation inactivated the TACSTD2 protein, changing the subcellular localization of the CLDN1 and CLDN7 proteins and thereby presumably disrupting the epithelial barrier function of the corneal epithelium.

## References

[1] Fujiki K, Nakayasu K, Kanai A (2001). Corneal dystrophies in Japan. J. Hum. Genet..

[2] Tsujikawa M (1998). Homozygosity mapping of a gene responsible for gelatinous drop-like corneal dystrophy to chromosome 1p. Am. J. Hum. Genet..

[3] Tsujikawa M (1999). Identification of the gene responsible for gelatinous drop-like corneal dystrophy. Nat. Genet..

[4] Malthiery Y, Lissitzky S (1987). Primary structure of human thyroglobulin deduced from the sequence of its 8448-base complementary DNA. Eur. J. Biochem..

[5] Yoshida S (2002). Two brothers with gelatinous drop-like corneal dystrophy at different stages of the disease: role of mutational analysis. Am. J. Ophthalmol..

[6] Ha NT, Fujiki K, Hotta Y, Nakayasu K, Kanai A (2000). Q118X mutation of M1S1 gene caused gelatinous drop-like corneal dystrophy in a family with gelatinous drop-like corneal dystrophy. Am. J. Ophthalmol..

[7] Ren Z (2002). Allelic and locus heterogeneity in autosomal recessive gelatinous drop-like corneal dystrophy. Hum. Genet..

[8] Fujiki K, Nakayasu K, Kanai A (2001). Corneal dystrophies in Japan. J. Hum. Genet..

[9] Tian X (2004). Compound heterozygous mutations of M1S1gene in gelatinous drop-like corneal dystrophy. Am. J. Ophthalmol..

[10] Alavi A (2007). Four mutations (three novel, one founder) in TACSTD2 among Iranian GDLD patients. Invest. Ophthalmol. Vis. Sci..

[11] Markoff A (2006). A novel TACSTD2 gene mutation in a Turkish family with a gelatinous drop-like corneal dystrophy. Mol. Vis..

[12] Taniguchi Y (2005). A novel missense mutation in a Japanese patient with gelatinous drop-like corneal dystrophy. Am. J. Opthalmol..

[13] Murakami A, Kimura S, Fujiki K, Fujimaki T, Kanai A (2004). Mutations in the membrane component, chromosome 1, surfacemarker1 (M1S1) gene in gelatinous drop-like corneal dystrophy. Jpn. J. Ophthalmol..

[14] Ha NT (2003). A novel mutation of M1S1 gene found in a Vietnamese patient with gelatinous drop-like corneal dystrophy. Am. J. Ophthalmol..

[15] Tasa G (2001). A novel mutation in the M1S1 gene responsible for gelatinous drop-like corneal dystrophy. Invest. Ophthalmol. Vis. Sci..

[16] Nakatsukasa M (2011). Two novel mutations of TACSTD2 found in three Japanese gelatinous drop-like corneal dystrophy families with their aberrant subcellular localization. Mol. Vis..

[17] Nakatsukasa M (2010). Tumor-associated calcium signal transducer2 is required for the proper subcellular localization of claudin1 and 7: implications in the pathogenesis of gelatinous drop-like corneal dystrophy. Am. J. Pathol..

[18] Kinoshita S, Kawasaki S, Kitazawa K, Shinomiya K (2012). Establishment of a human conjunctival epithelial cell line lacking the functional TACSTD2 gene (an American Ophthalmological Society thesis). Trans. Am. Ophthalmol. Soc..

[19] Ide T (2004). A spectrum of clinical manifestations of gelatinous drop-like corneal dystrophy in Japan. Am. J. Ophthalmol..

